# Plantar pressures and stabilometry effects of ischemic compression in Flexor digitorum brevis muscle Myofascial Trigger Point: A prepost study

**DOI:** 10.1371/journal.pone.0329734

**Published:** 2025-08-14

**Authors:** Eva María Martínez-Jiménez, Ricardo Becerro-de-Bengoa-Vallejo, Marta Elena Losa-Iglesias, Eduardo Pérez-Boal, Jorge Posada-Ordax, Anna Sánchez-Serena, Bibiana Trevissón-Redondo, María Benito-de-Pedro, Vicenta Martínez-Córcoles, Israel Casado-Hernández

**Affiliations:** 1 Departamento de Enfermería. Facultad de Enfermería Fisioterapia y Podología. Universidad Complutense de Madrid,; 2 Facultad de Ciencias de la Salud. Campus de Alcorcón. Universidad Rey Juan Carlos,; 3 Departamento de Enfermería y Fisioterapia. Facultad de Ciencias de Salud. Campus de Ponferrada. Universidad de León, León, Spain; 4 Faculty of Health Sciences at Manresa, University of Vic - Central University of Catalonia (UVic-UCC), Av. Universitària, 4-6, Manresa, Spain; 5 Grupo de Investigación en Fisioterapia y Salud (FYSA), Departamento de Fisioterapia, Facultad de Ciencias de la Salud-HM Hospitales, Universidad Camilo José Cela, Villanueva de la Cañada, Madrid, España; 6 Instituto de Investigación Sanitaria Hospitales HM, Madrid, España; 7 Department of Behavioral Sciences and Health, Miguel Hernandez University of Elche, San Juan de Alicante, Spain; University of Puerto Rico Medical Sciences Campus: Universidad de Puerto Rico Recinto de Ciencias Medicas, UNITED STATES OF AMERICA

## Abstract

**Background:**

Ischemic compression is a manual therapy that improves range of motion, pain and disability in Myofascial Pain Syndrome. Plantar foot pain is a common clinical entity that could be due to Flexor digitorum brevis trigger point. Effect on balance and plantar pressures after ischemic compression in Flexor digitorum brevis muscle trigger point have not been checked.

**Methods:**

Eighteen subjects (aged 25.06 + /- 5.51 years) with bilateral Flexor digitorum brevis latent or active myofascial trigger points were recruited. Study design: pre-post study. We measured three static footprint and stabilometry variables before and after ischemic compression for 90 seconds at bilateral Flexor digitorum brevis Myofascial Trigger Point. A Shapiro-Wilk test was performed to check normality. Comparison of related measures was done by paired T-test or Wilcoxon Range Test depending on whether the distribution was normal or non-normal. Significant differences were considered with p-value <0.05. All statistics were calculated with a 95% confidence interval. Reliability was also assessed with an Intraclass correlation coefficient (ICC) and Standard error measured (SEM) calculation.

**Results:**

Most variables have good to perfect reliability, with the exception of four variables which had moderate reliability and two variables which had only slight reliability. Reliable stabylometric variables included anteroposterior displacement of COP and surface with EO and EC. The footprint and stabilometry variables showed no significant differences after ischemic compression.

**Conclusions:**

Ischemic compression in the Flexor digitorum brevis muscle showed no significant differences in plantar pressures and stabilometry. Other techniques like dry needling indicated worsened balance effects. More studies are required to check significant changes. The results are important because they demonstrate a technique to treat FDB MTrP without repercussions on plantar pressure or balance. NCT06509347 (clinicalTrials.gov) initial release 7/7/24 and last release 28/7/24.

## Introduction

The assessment of foot plantar pressures using a platform is increasingly gaining acceptance within the clinical and scientific community [1].

An increase in peak plantar pressures during both static and dynamic standing is associated with diabetes [2]. Even in patients with diabetic claw or hammer toe deformities, peak plantar pressures are found in the hallux and the second to fifth metatarsal heads [3]. In conditions of ankle equinus, peak plantar pressures are located at the central forefoot [4]. In Sever’s disease, these pressures are in the rear foot [5]. Flatfoot displays peak plantar pressures at the second metatarsal head. Even foot deformities such as cavus foot, hallux valgus, and others show abnormal peak plantar pressures that are significantly higher compared to healthy subjects. Peak plantar pressures [3,5] have also been proven to increase the risk of ulceration in diabetic patients [4].

The analysis of the movement of plantar pressure centers while standing has proven to be a reliable measure of balance and postural control. Typically, a platform provides five or six quantitative variables. These measures are often referred to as stabilometry in research and are used to assess the effect of numerous treatments and conditions on balance [6–8].

Myofascial pain syndrome is a disorder characterized by a combination of sensory, motor, and autonomic symptoms. These symptoms typically result in referred pain that is unique for each muscle and is caused by trigger points [9,10].

Myofascial trigger points can be treated using various techniques and modalities. These include dry needling, local injection, ischemic compression, stretching, massage, and other methods [10,11]. Among these, dry needling or local injection, which physically stimulate trigger points, are efficient for Myofascial Pain Syndrome (MPS), as they reduce muscle shortening and increase blood flow [12].

The techniques based on mechanical stimulation include dry needling and ischemic compression [10]. Dry needling involves the insertion of an acupuncture needle into the trigger point to elicit more switch responses [11]. On the other hand, ischemic compression is a manual conservative treatment considered one of the most effective treatments for MPS [13]. It involves applying pressure on the myofascial trigger point for 90 s and progressing according to the patient’s tolerance. This stimulates the mechanoreceptors, which reduces pain signals and leads to the normalization of the biomechanical properties of the muscle fibers [13].

Dry needling and ischemic compression have proven to be effective treatments for MPS, reducing pain, increasing range of movement, and improving functionality [14,15]. In comparative studies, the primary difference noted between dry needling and ischemic compression is that the analgesic effect of dry needling is immediate. However, over the long term, the pain reduction achieved by both methods is equivalent [14].

Ischemic compression, also effective in the long term as dry needling, has been defined as a non-invasive alternative for the treatment of myofascial trigger points [16].

In one population-based study of 4,060 subjects, 17.4% experienced foot pain, aching, or stiffness in either foot [12].

Plantar heel pain is a common clinical issue that has a high incidence among the musculoskeletal pathologies of the foot. Approximately 10% of the population will suffer from MPS in their lifetime [17]. In the context of foot-related MPS, one of the causes of heel pain is the referred pain produced by the MPS of the Flexor digitorum brevis (FDB) [18].

The balance effect related to the treatment of MPS of the FDB muscle, therapeutic interventions of myofascial release, and dry needling, has been demonstrated to alter plantar pressures and balance [9,11]. It is shown that myofascial release in the foot’s plantar region enhances postural control by reducing the movement of the center of pressure [10], and it also increases surface and maximum pressure in the forefoot [19]. Dry needling in the FDB enhances the medium pressure on the midfoot and forefoot surfaces and simultaneously decreases maximum pressure in the rear foot. It also minimizes postural control after trials with eyes closed [18–20]. To date, there is only one study that investigates the long-term effects of combined treatment involving ischemic compression and joint mobilization for chronic foot pain. The study found that this combined therapy is associated with significant improvements in functionality and self-perceived improvement, both instantly after treatment and up to 6 months post-treatment [21].

Although ischemic compression is commonly used by clinicians on the foot [18], no study investigating the effects of ischemic compression on balance and plantar pressures has been conducted. We hypothesize that ischemic compression will reduce plantar pressures and improve balance following its application.

## Materials and methods

Eighteen subjects (aged 25.06 + /- 5.51 years) with bilateral Flex Flexor digitorum brevis or Digitorum Brevis latent or active myofascial trigger points were recruited. Study design: pre-post study. Patients were recruited at a private clinic. Those requiring ischemic compression treatment were invited to participate. Sampling continued consecutively until 18 participants were obtained. No participants were lost during the study. Socio-demographic variables between men and women were similar and did not result in any statistically significant differences.

The following inclusion criteria were applied: (1) All subjects experienced bilateral heel pain resulting from latent or active myofascial trigger points (MTrP) in the FDB muscle and did not have another active myofascial trigger point in the foot or lower limb; (2) Participants fell into the ‘normal weight’ category according to body mass index classifications, excluding those who are overweight or underweight [21]; (3) Participants were aged between 22 and 31 years.

Exclusion criteria: (1) Other pathologies of plantar pain associated such as plantar [21] fasciitis, tendinopathy, bursitis, sprains [22]; (2) Previous lower limb surgery [23]; (3) or plantar fasciitis treatment, tendinopathy, bursitis, sprains [8,22]; (4) Previous lower limb surgery [24,25] and previous treatment to their MTrP of Flexor digitorum brevis [25,26]; (4) diabetes mellitus [27]; foot digital deformity or congenital deformity [28]; (6) strenuous exercise or has drunk alcohol consumption for 24 h and/or consuming stimulants (e.g., caffeine) for 6 h prior to testing [23]; (7) any lower limb pathology in the past year and consented with pain; no evidence of a leg length discrepancy greater than 1 cm (from the anterior superior iliac spine to the upper surface of the most prominent aspect of the medial malleolus); have at least 15° of ankle dorsiflexion [22]; (8) no evidence of loss of balance using a validated test SEBT [22]; and (9) no loss of ankle dorsiflexion using modified lunge test [22].

The Ethics Committee of Rey Juan Carlos University granted its approval for the study under reference number 1912202200923. The consent form received approval from the ethics committee.

All subjects who met the inclusion and exclusion criteria were informed about the study procedure. Written consent was obtained, through the ethics committee, from all participants before the commencement of the procedure and intervention.

The ethical principles for medical research involving human subjects as stated in the Declaration of Helsinki, adopted at the 18th Assembly of the World Medical Association (WMA) in Helsinki, Finland, in June 1964, and later amended at the 52nd General Assembly in Edinburgh, Scotland, in October 2000, will be employed. Notable amendments include a clarification of paragraph 29 made at the WMA General Assembly in Tokyo, 2004, and the latest revisions from the 64th WMA General Assembly held in Fortaleza, Brazil, in October 2013 [29]. The study will be registered prospectively under the number NCT05836142 on ClinicalTrials.gov. The study is set to commence on 27-05-2023, with a primary completion date of 31-05-2023, and an overall study completion date of 2-06-2023.

### Sample size calculation

Using G*Power® 3.1.9.7 software, we calculated the necessary sample size to achieve the study’s objective. In examining a similar study by researchers investigating the acute effects of dry needling in the FDB muscle, it was discovered that there were significant differences in three stabilometric variables before and after the procedure. The surface with eyes closed changed (29.36–53.21 mm^2^, p = 0.000), as did the medium speed of the laterolateral displacement with eyes closed (1.42–1.64 mm/s, p = 0.004), and the medium speed of the anteroposterior displacement with eyes closed (1.30–1.53 mm/s, p = 0.025) [10,18].

Therefore, we performed an a priori sample size calculation for a t-family test, using differences between two dependent means. This approach applies to measures that are repeated in the same group, proceeding from a two-tailed hypothesis, a 95% statistical confidence level, an alpha error probability of 0.05, and a statistical power of 80% (with a beta error of 20%). We adopted an effect size of 0.52, which determined that a sample size of 18 subjects was required. In S1 Fig., you could see CONSROT Flow of research, how the enrolment has been done. No loss occurred.

### Procedures

Subjects seeking treatment for heel and/or plantar pain were examined. After confirming that their pain was due to the MTrP of the FDB muscle, and verifying that ischemic compression is the best method to treat MTrP of the FDB, we invited each subject to participate in the study.

Upon confirming participation, we verified all inclusion and exclusion criteria. Subsequently, participants were informed and signed the ethics committee agreement.

Protocol Study: (1) Initially, the registration order of three static footprints and three stabilometries each with eyes open (EO) and eyes closed (EC) was randomized. The record of each of these three conditions was randomized using free software at OxMaR®, Oxford Minimization, and Randomization during the two measurement intervals. (2) The first measurement was taken before the intervention. (3) Next, bilateral ischemic pressure treatment was applied. (4) This was followed by an immediate post-treatment evaluation which involved a new randomization of the same previous recordings.

Posture Registration: A barefoot protocol was performed on the platform, with the feet kept 30° to the midline. The posture was upright, and the upper limbs were relaxed, hanging naturally along the body for the recording of the static footprint and stabilometry. Each stabilometry recording lasted 30 s, during which the most upright position was maintained without any movement. The gaze was fixed on a point on the wall 2 m away. This practice was followed for both the recording of the static plantar footprint and stabilometry.

Intervention: The ischemic pressure technique was applied by positioning the thumbs at 90° to the muscle tissue. Continuous vertical pressure was applied following the methodology described by Fryer and Hodson, with the treatment extended to 90 s [30]. All measurements were taken before and after the ischemic pressure compression on the same day.

### Instrument

The instrument used is one of the few platforms validated in a specific investigation [8]. The static reliability studies demonstrate an Intraclass Correlation Coefficient from 0.873 to 0.975 for intrasession reliability, and for intersession reliability, a value from 0.879 to 0.966. These values correspond to perfect reliability according to the Landis and Koch criterion.

The reliability of stabilometry has been verified with the same posture used in this research, displaying intersession reliability ranging from 0.766 to 0.979 of Intraclass Correlation Coefficient (ICC) – a good reliability level as per Koo and Li [20,31], which is considered more than moderate, but rather substantial [20]. For the velocity of the center of pressure stabilometry variables, intrasession ICC values vary between 0.43 to 0.854, indicating a moderate to substantial level of reliability (20 acute effects). While there have been no studies conducted on intersession reliability, our study’s data was gathered in a single session.

We have also conducted a reliability study of our measures to ensure their reliability. No inter-observer reliability studies have been carried out in static or stabilometry using this platform.

As a technical specification platform, Podoprint® (Medicapteurs, Balma France) possesses 2304 sensors in a 400 × 400 mm configuration and has an acquisition frequency of 200 Hz. The system includes an automatic calibration feature before each trial. All technical characteristics are provided in Table 1. The software used in this context is Podoprint software, developed by Medicapteurs France SAS, Balma, France.

**Table 1:** Podoprint platform technical specifications.

Size (length _ width _ height)	530mm x 600 mm x 45 mm
Thickness	4 mm
Active surface	400 _ 400 mm
Weight	6.8 kg
Sensors Calibrated resistive	Calibrated resistive
Sensor’s area	8 x 8 mm
Sensors thickness	0.15 mm
Number of sensors	2304 (48x48)
Permissible temperature	40^o^ C to 85^o^ C
Sensor pressure	Minimum 0.0004 Kpa to 0.1 Kpa
Data acquisition frecuency	200 images/second
Vertical Forze recording	60 Hz

Abbreviations: mm (milimeters), Kg (kilograms), ^o^ C (degrees Celsius)), Kpa (Kilopascals), Hz(Hertz)

### Study variables

#### Static footprint.

The foot area was divided into three bilateral regions: (1) the bilateral rearfoot, (2) the bilateral midfoot, and (3) the bilateral forefoot (1). Medium Pressure Variable: This is the average force divided by the given plantar surface, measured in kilopascals per square centimeter; (2) Maximum Pressure Variable: This is the maximum force divided by the specified plantar surface, also measured in kilopascals per square centimeter. Besides these, we also calculated the Surface Variable, which measures the foot area in contact with the ground and receiving forces. This is computed in square centimeters.

#### Stabilometry variables.

All stabilometry variables are associated with the evaluation of Centre of Pressure (COP) movement. These variables include X displacement (mm), which refers to the distance traveled by the COP in the X axis within 30 s, and Y displacement (mm), referring to the similar measurement in the Y axis. The average latero-lateral displacement velocity (mm/s) is the mean velocity of the COP’s lateral movement within 30 s. The average anteroposterior displacement velocity (mm/s) depicts the mean velocity of the anterior-posterior movement of the COP in 30 s. Surface area (mm^2^) is the area covered by the COP in the same period.

All these variables were measured with both EO and EC. All measurements were taken in the same recruitment clinic.

### Statics

The Shapiro-Wilk test was utilized to assess the normality of the variables in a sample of fewer than 30 subjects [18]. A variable’s distribution was deemed normal if p > 0.05. Descriptive statistical analysis was performed using the mean ± standard deviation (SD) and a 95% Confidence Interval (CI). We employed the ICC to evaluate the reliability and intrasession repeatability of each variable. The values for interpreting the ICC data were derived from the authors Landis and Koch. An ICC of 0.20 or less signifies slight agreement; an ICC ranging from 0.21 to 0.40 indicates fair agreement; an ICC from 0.41 to 0.60 suggests a moderate level; an ICC between 0.61 and 0.80 presents a substantial level; and an ICC of 0.81 or higher, indicates near perfect agreement [32].

We could also include the criterion from Koo and Li regarding ICC, which states that ICC values less than 0.5, between 0.5 and 0.75, between 0.75 and 0.9, and greater than 0.90 are indicative of poor, moderate, good, and excellent reliability, respectively [31].

To assess the margin of error for each metric, the standard errors of the mean (SEM) were calculated. The SEM was determined between sessions using the ICCs and SD. The SEM is given as s_x.√(1 – r_xx), where s_x represents the SD of the observed test score set, and r_xx is the reliability coefficient for these data. In this context, the ICC is used to denote r_xx.

The minimum detectable change (MDC) was computed from the SEM using an established formula. Set at a 95% confidence level, this parameter signifies the extent of change necessary to ensure that any detected change is not due to mere random fluctuation or measurement error.

The MDC was also calculated at a 95% confidence level, thereby ensuring that any change in the measurement was not due to random variation or measurement error. This calculation was acquired from the SEM values employing the formula: MDC = √2 × 1.96 × SEM. Both the SEM and the MDC were analyzed following the method of Bland and Altman [33].

Additionally, the mean value of the three measurements for each variable was utilized. The Wilcoxon signed-rank test was conducted to identify any differences in non-parametric variables, and the paired t-test was applied to parametric variables both of them for comparison of related measures [10].

To ascertain the magnitude of this difference, we are required to compute Cohen’s D, as illustrated in Tables 3 and 4. This value is interpreted as follows: 0.2 represents a small effect size, 0.5 corresponds to a medium effect size, and 0.8 indicates a large effect size.

**Table 3:** Reliability and values on normality of all variables.

	Pre-test (*N* = 18)			Post-test (*N* = 18)		
Variable	ICC (95% CI)	SEM	Values of Normality 95% CI	ICC (95% CI)	SEM	Values of Normality 95% CI
Static foot plantar variables						
Rear Foot Maximum Pressure (K Pa)	0.978 (0.951-0.991)	4.08	89.43-132.77	0.989 (0.975-0.995)	2.83	91.36-139.64
Rear Foot Surface (cm^2^)	0.999 (0.998-1.00)	9.30	101.37-128.83	0.980 (0.957-0.992)	1.64	83.65-150.55
Midfoot Maximum Pressur (K Pa)	0.975 (0.945-0.990)	2.31	89.43-132.77	0.973 (0.941-0.989)	2.34	91.36-139.64
Midfoot Surface (cm^2^)	0.994 (0.987-0.998)	2.27	101.37-128.83	0.980 (0.956-0.992)	7.58	83.65-150.55
Fore foot Maximum Pressure (K Pa)	0.994 (0.987-0.998)	1.82	89.43-132.77	0.994 (0.986-0.997)	0.97	91.36-139.64
Fore Foot Surface (cm^2^)	0.999 (0.998-1.00)	52.49	101.37-128.83	0.976 (0.947-0.990)	12.44	83.65-150.55
Stabylometric variables						
X Displacement eyes open (mm)	0.803 (0.566-0.920)	0.77	89.43-132.77	0.686 (0.311-0.873)	1.91	91.36-139.64
Y Displacement eyes open (mm)	0.971 (0.936-0.988)	2.55	101.37-128.83	0.976 (0.947-0.990)	2.57	83.65-150.55
Surface eyes open(mm^2^)	0.276 (−0.590-0.707)	16.06	89.43-132.77	0.590 (0.101-0.834)	14.88	91.36-139.64
Medium Speed of the lateral Displacement Eyes open (mm/s)	0.760 (0.472-0.903)	0.137	101.37-128.83	0.751 (0.452-0.899)	0.13	83.65-150.55
Medium Speed of the anteroposterior Displacement eyes open (mm/s)	0.833 (0.633-0.932)	0.093	89.43-132.77	0.424 (0.264-0.767)	0.14	91.36-139.64
X Displacement eyes closed (mm)	0.772 (0.500-0.908)	7.84	101.37-128.83	0.845 (0.659-0.937)	0.13	83.65-150.55
Y Displacement eyes closed (mm)	0.976 (0.948-0.990)	0.20	89.43-132.77	0.985 (0.967-0.994)	0.71	91.36-139.64
Surface eyes closed (mm^2^)	0.485 (−0.130-0.792)	15.63	101.37-128.83	0.106 (−0.964-0.638)	20.49	83.65-150.55
Medium speed of the lateral displacement eyes closed (mm/s)	0.759 (0.471-0.903)	0.23	89.43-132.77	0.853 (0.676-0.940)	0.15	91.36-139.64
Medium speed of the anteroposterior displacement eyes closed (mm/s)	0.528 (−0.360-0.809)	0.33	101.37-128.83	0.829 (0.625-0.931)	0.12	83.65-150.55

Abbreviation: Kg (Kilograms); cm (centimeters), cm^2^ (centimeters^2^), SD (standard deviation), CI 95% (confidence interval 95%)

**Table 4:** Comparison of variables before and after ischemic compression.

	Ischemic compression Pre-Test (*N* = 18)		Ischemic compression Post-Test (*N* = 18)			Effect Size
Variable	Mean ± SD (95% CI)	Median (RI) (CI 95%)	Mean ± SD (95% CI)	Median (RI) (CI 95%)	*p*	Cohen’s D
Static foot plantar variables						
Rear Foot Maximum Pressure (K Pa)	105.03 ± 27.56^*^ (91.32–118.74)	111.04(26.33)	104.42 ± 27.02^*^ (90.99–117.86)	106.67 (18.71)	^a^ 0.349*	−0.317
Rear Foot Surface (cm^2^)	155.38 ± 294.38^*^ (8.99–301.78)	87.00 (12.50)	88.96 ± 11.61^*^ (83.18–94.73)	86.66 (15.00)	^a^ 0.983*	0.224
Midfoot Maximum Pressure (K Pa)	19.10 ± 14.62 (11.83–26.38)	19.81 (21)	21.61 ± 14.30 (14.50–28.72)	21.97 (15.58)	0.234^&^	0.759
Midfoot Surface (cm^2^)	33.35 ± 29.38^*^ (18.74–47.96)	33.16 (28.83)	44.35 ± 53.62^*^ (17.68–71.01)	23.00 (35.75)	^a^ 0.338^*^	−0.318
Fore foot Maximum Pressure (K Pa)	61.07 ± 23.60^*^ (49.34–72.81)	62.16 (23.47)	75.08 ± 12.54^*^ (68.84–81.32)	75.72 (19)	0.983^*^	0.076
Fore Foot Surface (cm^2^)	129.81 ± 118.27^*^ (70.99–188.62)	88.16(29.75)	115.20 ± 80.31^*^ (75.26–115.14)	89.66 (11.33)	^a^ 0.316*	0.3
Stabylometric variables						
X Displacement eyes open (mm)	6.67 ± 4.53 (4.42–8.93)	4.76(6.52)	7.65 ± 3.41 (5.95–9.35)	7.38 (3.41)	0.269^&^	−0.269
Y Displacement eyes open (mm)	31.04 ± 15.01 (23.57–38.51)	31.25 (23.57)	28.16 ± 16.62 (19.89–36.43)	25.93 (23.24)	0.625^&^	−0.117
Surface eyes open(mm^2^)	25.88 ± 18.88^*^ (17.49–32.28)	18.85 (31.22)	28.16 ± 23.24 (19.89–36.43)	25.93 (23.24)	^a^ 0.619^*^	−0.086
Medium Speed of the lateral Displacement Eyes open (mm/s)	1.39 ± 0.28 (1.25–1.53)	1.40 (0.37)	1.34 ± 0.28 (1.20–1.48)	1.35 (0.33)	0.097^*^	0.146
Medium Speed of the anteroposterior Displacement eyes open (mm/s)	1.07 ± 0.23 (0.95–1.18)	1.15 (0.40)	1.07 ± 0.19 (0.98–1.14)	1.10 (0.28)	0.913^&^	−0.026
X Displacement eyes closed (mm)	8.47 ± 16.44 (5.99–10.95)	8.15(7.80)	7.43 ± 5.10 (5.21–9.66)	7.93 (6.29)	0.306^*^	0.284
Y Displacement eyes closed (mm)	32.64 ± 14.11 (25.62–39.66)	36.05 (21.91)	32.63 ± 15.85 (24.75–40.52)	38.43 (25.90)	0.996^&^	−0.001
Surface eyes closed (mm^2^)	30.31 ± 21.78^*^ (19.48–41.15)	25.30 (22.68)	29.63 ± 15.25^*^ (22.04–37.21)	26.35 (21.68)	0.122*	−0.032
Medium speed of the lateral displacement eyes closed (mm/s)	1.66 ± 0.47 (1.42–1.90)	1.50 (0.76)	1.48 ± 0.36 (1.30–1.67)	1.41 (0.40)	0.070^&^	0.455
Medium speed of the anteroposterior displacement eyes closed (mm/s)	1.46 ± 0.49 (1.22–1.61)	1.26 (0.95)	1.28 ± 0.30 (1.13–1.44)	1.28 (0.54)	0.050^&^	0.498

Abbreviations: N (Newtons); SD, Standard Deviation; CI 95%, Confidence interval 95%; RI, Range interquartile; grades; a p value in from

^a^Wilcoxon,

^b^T-paired test p value < 0.05 with a confidence interval of 95% was considered statistically significant,

* Non parametric variables

& parametric variables.

## Results

The results obtained from the sample demographic variables reveal no significant differences between men and women in terms of age, weight, height, BMI, and shoe size is shown in Table 2.

**Table 2:** Socio-demographic characteristics of the sample population of ischemic compression Vs non-emission laser group.

Variable Total (36 feet in 18 participants)	Ischemic compression Mean±SD Median (RI) CI 95% (n = 18)	Ischemic compression Women Mean±SD Median (RI) CI 95% (n = 11)	Ischemic compression Men Mean±SD Median (RI) CI 95%(n = 7)	*P-value*
Age (years)	25.06 ± 5.51^*^ 23.50 (8) (22.31- 27.80)	27.39 ± 6.02^*^ 25 (11) (24.39- 30.39)	28.56 ± 5.32^*^ 27.00 (9) (26.75- 30.36)	0.494^a^
Weight (Kg)	63.94 ± 10.23 63 (14) (58.86- 69.03)	63.17 ± 9.23 61.50 (11) (58.58- 63.73)	64.69 ± 9.09 64.50 (11) (61.62-67.77)	0.628^b^
Height (cm)	169.61 ± 0.77 170.00 (12) (166.09- 173.13)	165.63 ± 83 168.50 (14) (161.70- 169.97)	168.58 ± 8.11 170.00 (13) (165.84- 171.33)	0.455^b^
BMI (Kg/m^2^)	22.12 ± 2.35 21.94 (2.72) (20.95- 23.29)	22.94 ± 2.74* 22.44 (4.16) (18.55- 25.68)	22.72 ± 2.36 22.65 (3.18) (21.92-23.52)	0.154^a^
Size of shoe	39.94 ± 2.22 40.00 (3.3) (38.83- 41.05)	39.28 ± 2.10 39.00(4.16) (38.23- 40.33)	40.16 ± 2.23 22.65 (4) (39.41- 40.92)	0.558^b^

Abbreviations: Kg (Kilograms); cm (centimeters), BMI Body Mass Index), SD (standard deviation), CI 95% (confidence interval 95%),

* non parametric variable,

^a^U Mann-Whitney,

^b^T-Student.

The reliability study of variables is shown in Table 3. To compare variables before and after ischemic compression, we conducted a Paired T-test and a Wilcoxon Rank test; the results can be viewed in Table 4.

## Discussion

The goal of our study was to assess the effects of ischemic compression on the plantar pressures of the foot and balance in the FDB muscle.

Our study exhibits excellent reliability in most variables. Only the Medium Speed of Anteroposterior Displacement with EO and EC, along with the surface variables with EO and closed, display moderate reliability. Consequently, we will not incorporate these results into our discussion or conclusions.

All footprint variables yield reliable results, indicating that plantar pressures and surface do not change their values following ischemic compression.

According to Koo and Li’s criteria [31], the ICC of our research indicates that the variables with sufficient reliability to consider after ischemic compression are Y displacement with EO, Y displacement with EC, and yet another Y displacement with EO. Significant differences were found in Y displacement with EO.

No greater displacement is produced after ischemic compression in the FDB muscle. This increase is associated with worsened balance control in the longitudinal or transversal axis of the feet. Current research on the FDB suggests that this muscle plays a role in controlling standing stability, due to the type of cross-sectional area, force levels, and contraction velocity. The FDB has a moderate cross-sectional area and force, thus it can sustain more durable contractions [34].

Our study indicates no significant effects in footprint and stabilometry variables in healthy subjects. However, this can grow in other pathological conditions like flat foot, cavus foot, and comorbid variables in neuropathies, Charcot foot, diabetic foot, fasciitis, etc. [10].

Further studies are necessary to determine if we could consider all variables to check if our results indicate a difference in balance regulation in COP depending on the axis.

Current research suggests that lumbar back pain results in reduced control of center pressures, thereby leading to decreased balance in athletes and middle-aged adults [32,35].

In cases of sub-chronic low back pain, improved balance and less pain were observed after dry needling [29]. The electromyographic evaluation measures both at rest and during isometric contraction improved post-application of dry needling [36]. This, in our opinion, could be the reason for the enhanced balance following dry needling in lumbar muscles.

However, the intrinsic musculature of the foot appears to behave differently in balance tasks compared to other muscles. The foot is considered a critical sensor within the vestibular system, playing a significant role in both static and dynamic balance [37,38]. The authors suggest that this could be why treatment results for MPS in the foot differ from those in other parts of the body.

Some studies have linked pain to worse postural control and poorer stabilometric values, with differences in balance impairment depending on the injured tissue [39]. One study on the recovery of balance and gait patterns indicated that an improvement in knee pain does not necessarily correlate with balance improvement, at least not for the succeeding six weeks [36]. Further research is necessary to comprehensively define the relationship between pain and balance loss.

When comparing our research with the results of other therapies applied to the foot, such as myofascial induction therapy, it is clear that these approaches can reduce pain pressure threshold in the same application area [40]. Myofascial induction therapy in the foot area has proven to enhance balance, an effect presumably due to the stimulation of proprioceptors within the fascia. To date, based on our research, it is the only manual technique applied to the foot that has been shown to enhance balance. Meanwhile, the dry needling technique of the FDB has been associated with a loss of balance, noticeably more so in women than in men [41]. In our study, ischemic pressure did not result in a loss of equilibrium.

The results of static foot plantar pressures after dry needling Myofascial FDB revealed an increase in surface area, without any additional forefoot maximum pressure. We found an increase in medium maximum pressure across all medium pressure and forefoot areas.

In comparing the effects of dry needling with other therapies used on the FDB myofascial trigger point, we observed varied results. Dry needling results in an increase in the surface area of the plantar footprint static variables of the FDB, without raising the maximum forefoot pressure. Ischemic compression, meanwhile, has been shown to immediately reduce pain post-application [42]. It also causes no changes in plantar pressures and surface, so this intervention do not increase the maximum midfoot and forefoot pressures, these data serve as a no increase of risk factor for ulceration in patients with diabetes, so there are no detrimental effects applying ischemic compression in these patients [43]. Therefore, in treating the myofascial trigger point of the FDB muscle in metatarsalgia, ischemic compression will be not the best option, myofascial release and dry needling will be preferable. Preventively, the use of high heels and prolonged standing should be prevented as these factors contribute to metatarsalgia [44], and high-impact sports should be abstained from after treatment [45]. Dry needling should be considered. For contraindicated cases such as those on anticoagulants and diabetics [46,47], the use of ischemic pressure in alliance with padding or orthopedic unloading treatment should be evaluated, as it has been demonstrated to improve symptoms [45], along with other orthopedic treatments like the Morton extension, which reduces plantar pressures [48]. Recent research recommends the combined usage of both techniques, ischemic compression and dry needling to enhance effectiveness on myofascial pain syndrome- it is more advantageous than utilizing them separately [49]. But in consideration with balance effects ischemic compression will be more indicated in persons with a risk of falling because other techniques like dry needling have showed worsen balance effects. Our results are important because show a technique to treat FDB MTrP without plantar pressures and balance repercussion.

Further studies are necessary to compare various techniques, assess their impact thoroughly, and evaluate the effectiveness of combined techniques on plantar pressures and stabilometry. Moreover, it is pertinent to distinguish the balance of each technique according to sex and observe their long-term effects.

### Limitations

A larger sample size, including both men and women, would have facilitated a separate evaluation of the results by gender. However, we acknowledge that this aim is challenging, as it requires patients who have trigger points in the FDB of both feet. Future research should investigate potential differences between genders.

Additionally, more studies need to check if the effects on balance and plantar pressures are more significant in subjects with multiple comorbid factors, such as diabetes, old age, obesity, etc. A randomized clinical trial could provide better evidence results.

## Conclusion

No significant differences were found in static and stabilometry variables after ischemic compression in healthy subjects.

Ischemic compression in the Flexor digitorum brevis muscle showed no significant differences in plantar pressures and stabilometry. Other techniques like dry needling indicated worsened balance effects. More studies are required to check significant changes and in comorbid factors and diseases. The results are important because they demonstrate a technique to treat FDB MTrP without repercussions on plantar pressure or balance.

## Supporting information

S1 Fig CONSORT Flow diagram. (TIFF)

S2 File Clinical trial protocol. (PDF)

S3 File TREND-list. (PDF)

S4 File Data. (XLSX)
