# Supplementary figures and images for: Plantar pressures and stabilometry effects of ischemic compression in Flexor digitorum brevis muscle Myofascial Trigger Point: A prepost study
Source: PLoS One. 2025 Aug 14;20(8):e0329734. doi: 10.1371/journal.pone.0329734 (PMC12352831; doi:10.1371/journal.pone.0329734)

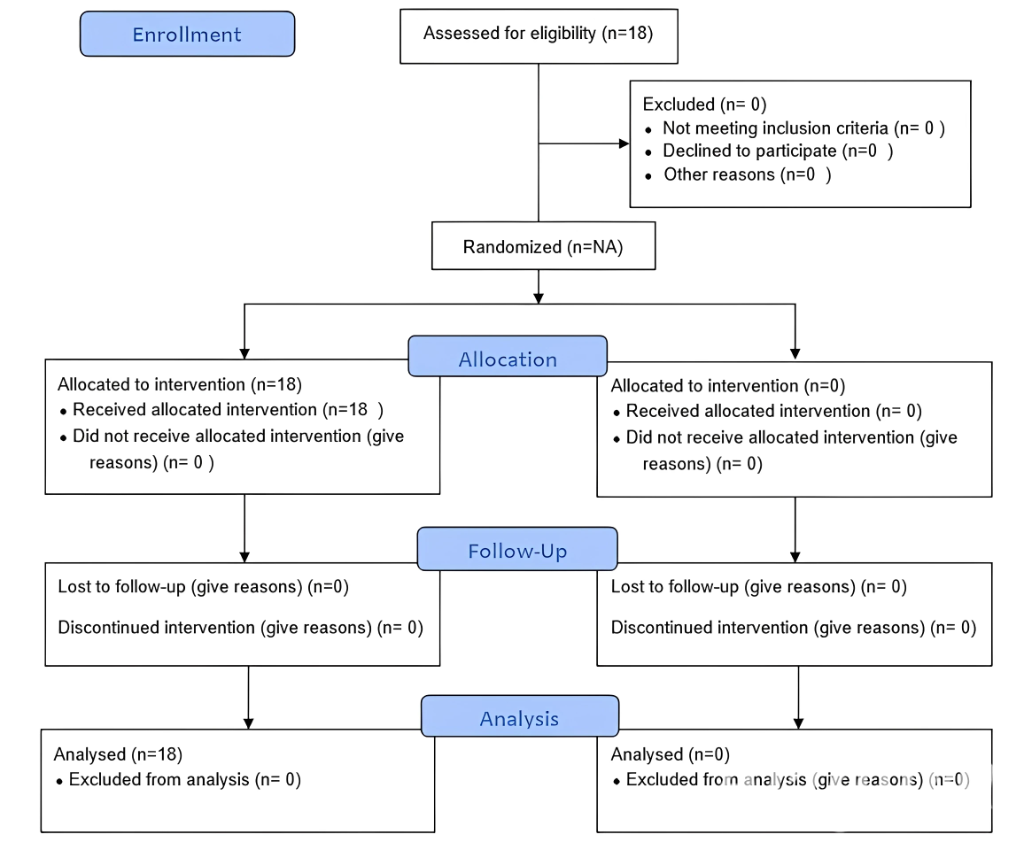

Supplement: S1 Fig — (TIFF) [file pone.0329734.s001.tiff]
