# Supplementary material for: Plantar pressures and stabilometry effects of ischemic compression in Flexor digitorum brevis muscle Myofascial Trigger Point: A prepost study
Source: PLoS One. 2025 Aug 14;20(8):e0329734. doi: 10.1371/journal.pone.0329734 (PMC12352831; doi:10.1371/journal.pone.0329734)
Supplement: S2 File — (PDF) [file pone.0329734.s002.pdf]

STUDY RPOTOCOL.

Plantar pressures and stabilometry effects of ischemic compression in Flexor digitorum brevis muscle Myofascial Trigger Point. A prepost study.

1.Study Description

Brief Summary: In this study we will test the effects of pressure release on the Flexor digitorum brevis muscle on stabilometric variables that support static equilibrium.

Detailed Description: In this study we will test the effects of pressure release on the Flexor digitorum brevis muscle on the stabilometric variables that support static balance. To test the effects on balance.

2.Conditions

Conditions: Balance; plantar pressures; Manual Therapy; Foot; Myofascial Pain Syndrome; Myofascial Trigger Point

Keywords: balance; myofascial trigger point; foot

3.Study Design

Study Type: Interventional

Primary Purpose: Treatment

Interventional Study Model

Number of Arms: 1

Allocation: Randomized

Subjects

Eighteen subjects (aged 25.06+/- 5.51 years) with bilateral Flex Flexor digitorum brevis or Digitorum Brevis latent or active myofascial trigger points were recruited. Study design: pre-post study. Patients were recruited at a private clinic. Those requiring ischemic compression treatment were invited to participate. Sampling continued consecutively until 18 participants were obtained.

4. Arms and Interventions

Experimental: Pressure release

Application of pressure on the trigger point of the flexor digitorum brevis until remission of referred pain for a minimum of 90 seconds, at least 3 cycles.

Bilateral non emission Laser in latent trigger points of the Flexor digitorum Brevis Muscle

Outcome Measures

Primary Outcome Measure:

1. Stabilometric variable x displacement open eyes before pressure release.

Stabilometry assessment was used and subjects were instructed to stand barefoot on the force platform, participants were instructed to remain in a relaxed standing posture with feet shoulder-width apart and positioned at 30º away from the midline. Stabilometry variables measure displacement of the center of pressures in X (in millimeters) with open eyes.

[Time Frame: Through study completion, an average of 1 week.]

2. Stabilometric variable center of pressure area open eyes before pressure release.

Stabilometric assessment was used and subjects were instructed to stand barefoot on the force platform, participants were instructed to remain in a relaxed standing posture with feet shoulder-width apart and positioned at 30º away from the midline. Stabilometric variables measure displacement of the center of Pressure (COP) area (in $millimeters^2$) with eyes open.

[Time Frame: Through study completion, an average of 1 week.]

3. Stabilometric variable center of pressure speed displacement of the anteroposterior(a-p) direction open eyes before pressure release.

Stabilometric assessment was used and subjects were instructed to stand barefoot on the force platform, participants were instructed to remain in a relaxed standing posture with feet shoulder-width apart and positioned at 30º away from the midline. Stabilometric variables measure center of pressure speed displacement of the anteroposterior(a-p) direction (in millimeters/second).

[Time Frame: Through study completion, an average of 1 week.]

6.Stabilometric variable y displacement open eyes before pressure release.

Stabilometric assessment was used and subjects were instructed to stand barefoot on the force platform, participants were instructed to remain in a relaxed standing posture with feet shoulder-width apart and positioned at 30º away from the midline. Stabilometric variables measure displacement of the center of pressures in Y (in millimeters) with open eyes.

[Time Frame: Through study completion, an average of 1 week.]

8. Stabilometric variable center of pressure speed displacement of the latero-lateral(lat-lat) direction open eyes before pressure release.

Stabilometric assessment was used and subjects were instructed to stand barefoot on the force platform, participants were instructed to remain in a relaxed standing posture with feet shoulder-width apart and positioned at 30º away from the midline. Stabilometry variables measure center of pressure speed displacement of the latero-lateral(lat-lat) direction (in millimeters/second).

[Time Frame: Through study completion, an average of 1 week.]

9. Stabilometric variable x displacement closed eyes before pressure release.

Stabilometric assessment was used and subjects were instructed to stand barefoot on the force platform, participants were instructed to remain in a relaxed standing posture with feet shoulder-width apart and positioned at 30º away from the midline. Stabilometric variables measure displacement of the center of pressures in X (in millimeters) with closed eyes.

[Time Frame: Through study completion, an average of 1 week.]

10.Stabilometric variable center of pressure area closed eyes before pressure release.

Stabilometric assessment was used and subjects were instructed to stand barefoot on the force platform, participants were instructed to remain in a relaxed standing posture with feet shoulder-width apart and positioned at 30º away from the midline. Stabilometric variables measure displacement of the center of Pressure (COP) area (in millimeters^2) with closed eyes.

[Time Frame: Through study completion, an average of 1 week.]

14. Stabilometric variable center of pressure speed displacement of the anteroposterior(a-p) direction closed eyes before pressure release.

Stabilometric assessment was used and subjects were instructed to stand barefoot on the force platform, participants were instructed to remain in a relaxed standing posture with feet shoulder-width apart and positioned at 30º away from the midline. Stabilometric variables measure center of pressure speed displacement of the anteroposterior(a-p) direction (in millimeters/second).

[Time Frame: Through study completion, an average of 1 week.]

15.Stabilometric variable y displacement closed eyes before pressure release.

Stabilometric assessment was used and subjects were instructed to stand barefoot on the force platform, participants were instructed to remain in a relaxed standing posture with feet shoulder-width apart and positioned at 30º away from the midline. Stabilometric variables measure displacement of the center of pressures in Y (in millimeters) with closed eyes.

[Time Frame: Through study completion, an average of 1 week.]

18. Stabilometric variable center of pressure speed displacement of the latero-lateral(lat-lat) direction closed eyes before pressure release.

Stabilometric assessment was used and subjects were instructed to stand barefoot on the force platform, participants were instructed to remain in a relaxed standing posture with feet shoulder-width apart and positioned at 30º away from the midline. Stabilometric variables measure center of pressure speed displacement of the latero-lateral(lat-lat) direction (in millimeters/second).

[Time Frame: Through study completion, an average of 1 week.]

20. Variables of plantar pressures with platform before pressure release

For 30 seconds we will record the plantar pressure variables in grams per square centimetre.

[Time Frame: Through study completion, an average of 2 days]

21. Variable footprint planting surface with platform before non-emission laser For 30 seconds we will record the area of the footprint in square centimetres.

[Time Frame: Through study completion, an average of 2 days]

22.. Variable footprint plantar surface with platform before pressure release For 30 seconds we will record the plantar pressure variables in grams per square centimetre.

[Time Frame: Through study completion, an average of 2 days]

5. Variables of plantar pressures with platform after preassure release.

For 30 seconds we will record the plantar pressure variables in grams per square centimetre.

[Time Frame: Through study completion, an average of 2 days]

7. Variable footprint planting surface with platform after pressure release For 30 seconds we will record the area of the footprint in square centimetres.

[Time Frame: Through study completion, an average of 2 days]

Eligibility

Minimum Age: 18 Years

Maximum Age: 39 Years

The following inclusion criteria were applied: (1) All subjects experienced bilateral heel pain resulting from latent or active myofascial trigger points (MTrP) in the FDB muscle and did not have another active myofascial trigger point in the foot or lower limb; (2) Participants fell into the 'normal weight' category according to body mass index classifications, excluding those who are overweight or underweight; (3) Participants were aged between 22 and 31 years.

Exclusion criteria: (1) Other pathologies of plantar pain associated such as plantar fasciitis, tendinopathy, bursitis, sprains ; (2) Previous lower limb surgery ; (3) or plantar fasciitis treatment, tendinopathy, bursitis, sprains ; (4) Previous lower limb surgery and previous treatment to their MTrP of Flexor digitorum brevis ; (4) diabetes mellitus ; foot digital deformity or congenital deformity ; (6) strenuous exercise or has drunk alcohol consumption for 24 h and/or consuming stimulants (e.g. caffeine) for 6 h prior to testing ; (7) any lower limb pathology in the past year and consented with pain; no evidence of a leg length discrepancy greater than 1 cm (from the anterior superior iliac spine to the upper surface of the most prominent aspect of the medial malleolus); have at least 15° of ankle dorsiflexion; (8) no evidence of loss of balance using a validated test SEBT ; and (9) no loss of ankle dorsiflexion using modified lunge test

The Ethics Committee of Rey Juan Carlos University granted its approval for the study under reference number 1912202200923

Protocol Study

(1) Initially, the registration order of three static footprints and three stabilometries each with eyes open (EO) and eyes closed (EC) was randomized. The record of each of these three conditions was randomized using free software at OxMaR®, Oxford Minimization, and Randomization during the two measurement intervals. (2) The first measurement was taken before the intervention. (3) Next, bilateral ischemic pressure treatment was applied. (4) This was followed by an immediate post-treatment evaluation which involved a new randomization of the same previous recordings.

Posture Registration: A barefoot protocol was performed on the platform, with the feet kept 30° to the midline. The posture was upright, and the upper limbs were relaxed, hanging naturally along the body for the recording of the static footprint and stabilometry. Each stabilometry recording lasted 30 s, during which the most upright position was maintained without any movement. The gaze was fixed on a point on the wall 2 m away. This practice was followed for both the recording of the static plantar footprint and stabilometry.

Intervention: The ischemic pressure technique was applied by positioning the thumbs at 90° to the muscle tissue. Continuous vertical pressure was applied following the methodology described by Fryer and Hodson, with the treatment extended to 90 s [32]. All measurements were taken before and after the ischemic pressure compression on the same day

Contacts/Locations

Central Contact Person: EVA MARÍA MARTÍNEZ JIMENEZ, PhD

Telephone: +34616255378

Email: eva.hache2@hotmail.com

Central Contact Backup:

Study Officials: NOTE : Study Official is required by the WHO and ICMJE.

Locations: **Spain**

Eva María Martínez-Jiménez In Mayuben CLinic

Madrid, Spain, 28702

Contact: Eva María MARIA Martínez-Jiménez +34616255378

evamam03@ucm.es

NOTE : Contact Middle Initial should have no more than 2 characters
